# Root respiratory burst oxidase homologue-dependent H_2_O_2_ production confers salt tolerance on a grafted cucumber by controlling Na^+^ exclusion and stomatal closure

**DOI:** 10.1093/jxb/erx386

**Published:** 2017-11-14

**Authors:** Mengliang Niu, Yuan Huang, Shitao Sun, Jingyu Sun, Haishun Cao, Sergey Shabala, Zhilong Bie

**Affiliations:** 1 College of Horticulture and Forestry, Huazhong Agricultural University and Key Laboratory of Horticultural Plant Biology, Ministry of Education, Wuhan, P. R. China; 2 School of Land and Food, University of Tasmania, Hobart, Tasmania, Australia

**Keywords:** Grafting, H^+^-ATPase, Na^+^ exclusion, ROS, salinity, signaling, stomatal closure

## Abstract

Plant salt tolerance can be improved by grafting onto salt-tolerant rootstocks. However, the underlying signaling mechanisms behind this phenomenon remain largely unknown. To address this issue, we used a range of physiological and molecular techniques to study responses of self-grafted and pumpkin-grafted cucumber plants exposed to 75 mM NaCl stress. Pumpkin grafting significantly increased the salt tolerance of cucumber plants, as revealed by higher plant dry weight, chlorophyll content and photochemical efficiency (*F*_v_/*F*_m_), and lower leaf Na^+^ content. Salinity stress resulted in a sharp increase in H_2_O_2_ production, reaching a peak 3 h after salt treatment in the pumpkin-grafted cucumber. This enhancement was accompanied by elevated relative expression of respiratory burst oxidase homologue (RBOH) genes *RbohD* and *RbohF* and a higher NADPH oxidase activity. However, this increase was much delayed in the self-grafted plants, and the difference between the two grafting combinations disappeared after 24 h. The decreased leaf Na^+^ content of pumpkin-grafted plants was achieved by higher Na^+^ exclusion in roots, which was driven by the Na^+^/H^+^ antiporter energized by the plasma membrane H^+^-ATPase, as evidenced by the higher plasma membrane H^+^-ATPase activity and higher transcript levels for *PMA* and *SOS1*. In addition, early stomatal closure was also observed in the pumpkin-grafted cucumber plants, reducing water loss and maintaining the plant’s hydration status. When pumpkin-grafted plants were pretreated with an NADPH oxidase inhibitor, diphenylene iodonium (DPI), the H_2_O_2_ level decreased significantly, to the level found in self-grafted plants, resulting in the loss of the salt tolerance. Inhibition of the NADPH oxidase-mediated H_2_O_2_ signaling in the root also abolished a rapid stomatal closure in the pumpkin-grafted plants. We concluded that the pumpkin-grafted cucumber plants increase their salt tolerance via a mechanism involving the root-sourced respiratory burst oxidase homologue-dependent H_2_O_2_ production, which enhances Na^+^ exclusion from the root and promotes an early stomatal closure.

## Teaser

Root respiratory burst oxidase homologue-dependent H_2_O_2_ production serves as an early signal to confer salt tolerance on a grafted cucumber through regulation of Na^+^ exclusion and stomatal closure.

## Introduction

Soil salinity is a global challenge affecting agricultural production worldwide. More than 800 million hectares of agricultural land suffers from soil salinity (Rengasamy, 2010). Among all types of salinity, the most soluble and widespread salt is NaCl, and Na^+^ toxicity therefore prevails in most natural habitats restricting plant growth. For most glycophytes, the ability of a plant to minimize accumulation of the toxic Na^+^ in the sensitive shoot is a crucial feature of salinity tolerance. However, as most of the Na^+^ delivered to the shoot remains in the shoot, and only a small portion can be recirculated back to the root via the phloem (Munns and Tester, 2008), the salt tolerance largely depends on the capacity of plants to limit the net transport of Na^+^ from the root to the shoot. This process relies on several key mechanisms; one of them is the efficient Na^+^ efflux from the root to the external medium.

Active Na^+^ extrusion is mediated by the plasma membrane (PM) Na^+^/H^+^ antiporter (Shi *et al.*, 2002). Here, energy-dependent Na^+^ transport is coupled to the H^+^ electrochemical potential difference established by H^+^ translocation pumps (Hasegawa *et al.*, 2003). The NaCl-induced activation of the PM Na^+^/H^+^ antiporter has been reported in various plant species, such as tomato (Wilson and Shannon, 1995), Arabidopsis (Qiu *et al.*, 2002) and rice (Martínez-Atienza and Quintero, 2007).

Salt stress also induces the production of reactive oxygen species (ROS) (Zhen *et al.*, 2011). When accumulated in excessive quantities, ROS may react with various cellular targets such as nucleic acids, proteins, lipids and chlorophyll, causing serious damage (Niu and Liao, 2016). At the same time, besides their harmful effects, ROS can also act as signaling molecules that regulate plant development, and biotic and abiotic stress responses (Mittler *et al.*, 2004; Bose *et al.*, 2014; Li *et al.*, 2014; Li *et al.*, 2016). More and more evidence has accumulated suggesting that ROS play an important role in plant salinity tolerance (Wang *et al.*, 2013; Hossain and Dietz, 2016). For example, Arabidopsis *AtrbohF* knockout mutants, which lack the respiratory burst oxidase proteins (NADPH oxidases that catalyse the production of ROS in the apoplast), showed an increased salt sensitivity and impaired Na^+^/K^+^ homeostasis (Ma *et al.*, 2012; Jiang *et al.*, 2013). Among all the ROS, H_2_O_2_ has a comparatively long lifespan and a small size, which permits it to traverse the cellular membranes to different cellular compartments. Some recent findings led to speculation that H_2_O_2_ may act as a stress signal that regulates the PM Na^+^/H^+^ antiport system under saline conditions and alters *SOS1* mRNA stability in Arabidopsis which is fundamental to maintaining cellular K^+^/Na^+^ homeostasis (Zhang *et al.*, 2007).

H_2_O_2_ has also been demonstrated to mediate rapid systemic signaling stimulated by a root-derived ABA triggered by high temperature stress (Li *et al.*, 2014). However, the role and specific mechanisms of H_2_O_2_-induced root-to-shoot communication are largely unknown for salinity stress. Several papers demonstrated that H_2_O_2_ functions in the regulation of stomatal aperture (Desikan *et al.*, 2005; Danquah *et al.*, 2014; Niu and Liao, 2016). Silencing *RBOH1* led to an impaired capacity for stomatal closure in tomato (Zhou *et al.*, 2014; Yi *et al.*, 2015). However, all these reports dealt with H_2_O_2_ produced in (or applied to) the shoot and, to the best of our knowledge, no reports are available linking root-originating ROS signals with stomatal operation in salt-grown plants.

Grafting is a widely used agronomic practice that improves a plant’s salt tolerance by replacing the sensitive root with one taken from a more tolerant genotype or species. Plants of the Cucurbitaceae such as melon, watermelon, and cucumber are glycophytes of high economic importance, but all of them are sensitive to Na^+^ (Zhu *et al.*, 2008). At the same time pumpkin, which belongs to the same family, is considerably more tolerant under saline conditions (Lee *et al.*, 2010; Rouphael *et al.*, 2012). Grafting cucumber scion onto pumpkin rootstock can therefore potentially lead to higher salt tolerance in this species. Previous studies have suggested that pumpkin exhibited a higher capacity in limiting the transport of Na^+^ from root to shoot than melon and cucumber (Edelstein *et al.*, 2011; Huang *et al.*, 2013). Electrophysiological studies have also demonstrated that pumpkin roots exhibited a high efficiency in extruding Na^+^ (Lei *et al.*, 2014). As this increased extrusion was concurrent with an increased H^+^ influx under NaCl stress, this suggested that Na^+^ exclusion in salt stressed pumpkin roots was the result of an active Na^+^/H^+^ antiporter across the PM fueled by the plasma membrane H^+^-ATPase encoded by *PMA* (Li *et al.*, 2015). Na^+^/H^+^ exchange in the root was inhibited by amiloride (a Na^+^/H^+^ antiporter inhibitor) and vanadate (a PM H^+^-ATPase inhibitor) indicating that the H^+^-ATPase-driven Na^+^/H^+^ antiport plays an important role in dealing with salt stress (Sun *et al.*, 2009).

In the present study, we have compared the accumulation patterns of H_2_O_2_ and Na^+^ between two grafted combinations (self-grafted and pumpkin-grafted cucumber seedlings). The ion fluxes in root and hypocotyl were evaluated by the non-invasive micro-test technology (NMT). Linked with pharmacological experiments, these results demonstrate that root respiratory burst oxidase homologue (RBOH)-dependent H_2_O_2_ production confers salt tolerance on grafted cucumber by controlling Na^+^ exclusion and stomatal closure, thus optimizing plant ionic and water balance under hostile saline conditions.

## Materials and methods

### Grafting method and growth conditions

The experiment was carried out in the growth chambers at Huazhong Agricultural University, Central China. A salt-sensitive cucumber (*Cucumis sativus* L.) cv. Jinchun No. 2 (abbreviated here as ‘C’) was used, either as a scion or a rootstock, and a salt-tolerant pumpkin (*Cucurbita moschata* Duch.) cv. Chaojiquanwang (abbreviated as ‘P’) was used as a rootstock. Two grafted combinations were used in this study: cucumber self-grafted plants (C/C) and pumpkin-grafted plants (C/P). We did not use ungrafted plants as additional controls, since our previous studies showed that the response of ungrafted and self-grafted cucumber/pumpkin to salt stress was similar (Huang *et al.*, 2013); this included plant growth reduction, Na^+^ concentration, and stomatal conductance under salt stress. Thus, it was concluded that the advantage of grafted cucumber plants is attributable to the rootstock, not the grafting process itself (Lei *et al.*, 2014).

The seeds were soaked in tap water for 6 h and incubated in the dark at 30 °C until germination. Rootstocks were sown 4 d earlier than cucumber scions. When the rootstock seedlings had developed one true leaf, the cucumber seedlings were grafted onto them by using the ‘hole insertion grafting’ method described by Lee (1994). Briefly, the first true leaf of the rootstock was removed and the apex of the rootstock was perforated. The scion was prepared with two cuts giving a sharp edge of about 10 mm of hypocotyl. The scion was then inserted into the rootstock hole from the top. After grafting, the seedlings were placed in a ‘healing chamber’ in which the relative humidity was kept at ≥95% for the first 3 d and then gradually decreased to 75%. The air temperature was kept 28–30 °C, and plants were kept in the darkness for the first 48 h, and then exposed to a 14/10 h light/dark cycle, 28/18 °C, with photosynthetic photon ﬂux density 600 μmol m^–2^ s^–1^. After 7 d the grafted plants were transferred to plastic containers (six seedlings per container) containing 8 liters of full-strength Hoagland’s solution. The nutrient solution was refreshed at 3 d intervals and continuously aerated. At the four-leaf stage, grafted combinations were used for subsequent experiments.

### Salt treatment and NADPH oxidase inhibitor application

To study the Na^+^ and H_2_O_2_ accumulation patterns in two grafted combinations, NaCl was added into the growth media to obtain a final concentration of 75 mM. The choice of this specific concentration was determined by the fact that we aimed to investigate the signaling role of H_2_O_2_ and thus tried to select the concentration that was strong enough to reveal the phenotypic difference but could be considered ‘safe’ in terms of damage to the root. The time courses of malonyldialdehyde (MDA), relative electrical conductivity (REC), Na^+^ and H_2_O_2_ contents were monitored by plant sampling at 0, 1, 3, 12, 24, 48, and 120 h after commencement of salt treatment. The biomass, relative chlorophyll content (measured with a SPAD meter) and chlorophyll fluorescence (*F*_v_/*F*_m_) were measured 120 h after salt treatment.

It was true that 100 mM NaCl treatment led to a more obvious difference of the phenotype (see Supplementary Fig. S1 at *JXB* online), but high concentrations of NaCl (100 mM or higher) inevitably caused serious damage in the root of C/C with an enhanced H_2_O_2_ level (Supplementary Fig. S2). This increased H_2_O_2_ level was detected after 5 d of NaCl treatment, which might be a result of an impaired redox system rather than a signal. The purpose of this study was to evaluate the function of root-sourced H_2_O_2_ as a molecular signal, so we use 75 mM NaCl to distinguish the salt tolerance between two grafted combinations.

In some experiments, the NADPH oxidase inhibitor diphenylene iodonium (DPI) was added to the medium to a final concentration of 20 μM. The plants were pretreated with DPI for 6 h, and then transferred to Hoagland’s solution containing 75 mM NaCl. The treatments without DPI or NaCl were set as controls. H_2_O_2_ content, transpiration rate, stomatal conductance, NADPH oxidase activity, H^+^-ATPase activity, and related gene (*RbohD*, *RbohF*, *PMA*, *SOS1*) expression levels were determined 3 h after salt treatment. The tissue Na^+^ content and Na^+^ and H^+^ fluxes in roots and hypocotyls were determined 24 h after salt treatment.

### Relative chlorophyll content (SPAD) and chlorophyll fluorescence measurements

Relative chlorophyll content was measured with a chlorophyll meter (SPAD-502, Minolta Corp., Ltd, Osaka, Japan) from the fully expanded functional leaves (the third from the apex). Measurements were made at a central point on the leaflet between the midrib and the leaf margin. Chlorophyll fluorescence was determined with an imaging-PAM chlorophyll fluorometer (Heinz Walz, GmbH, Effeltrich, Germany). Plants were dark-adapted for 30 min to measure the maximum photochemical efficiency of PSII (*F*_v_*/F*_m_) at the same position as chlorophyll content.

### Analysis of lipid peroxidation and membrane permeability in leaves

The level of lipid peroxidation in leaves was assessed by measuring the content of malondialdehyde (MDA) using the thiobarbituric acid reaction (Heath and Packer, 1968). Membrane permeability of the leaf was measured as the relative electrical conductivity according to the method described by Zhou and Leul (1998). The washed leaves (0.1 g) were cut into 1 cm^2^ pieces and placed in a 50 ml test tube containing 30 ml deionized water. The leaf samples were immersed and vibrated for 3 h, and then the conductivity of the solution was measured using a conductivity meter (SG78, Mettler Toledo). After boiling the samples for 15 min, their conductivity was measured again when the solution was cooled to room temperature. The relative electrical conductivity (REC) was calculated as follows:

$$\text{REC}\left(\%\right)=\left(C_{\text{1}}/C_{\text{2}}\right)\times \text{1}00$$

where *C*_1_ and *C*_2_ are the electrolyte conductivities measured before and after boiling, respectively.

### Determination of H_2_O_2_ concentration in roots and leaves

H_2_O_2_ was extracted from 0.5 g fresh leaf or root samples ground in 3 ml of 1 M HClO_4_. After centrifugation, the supernatant was adjusted to pH 6.0–7.0 and filtered through a Sep-Pak C18 cartridge (Millipore, Milford, MA, USA). After elution with 4 ml distilled water, an aliquot of the sample (800 μl) was mixed with 400 μl reaction buffer containing 4 mM 2,2′-azino-di(3-ethylbenzthiazoline-6-sulfonic acid) and 100 mM potassium acetate at pH 4.4, and 400 μl deionized water. The reaction was started by the addition of 3 μl (0.5 U) of horseradish peroxidase. H_2_O_2_ content was measured spectrophotometrically at the optical density at 412 nm (Willekens *et al.*, 1997).

### Determination of Na^+^ content in roots and leaves

Dried roots and leaves of two grafted combinations were ground using a mortar and pestle; 0.1 g of powder was then digested with 5 ml of nitric acid for 3 h, and then Na^+^ concentrations were analysed using an atomic absorption spectrophotometer (Varian spectra AA 220, Varian, Palo Alto, CA, USA).

### Measurement of ion fluxes in roots and hypocotyls with NMT

A so-called ‘recovery protocol’ (Cuin *et al.*, 2011) was used to quantify the activity of the Na^+^ efflux system in plant root and hypocotyls. For this, net Na^+^ and H^+^ fluxes were measured using the non-invasive micro-test technology (NMT) technique (YoungerUSA LLC, Amherst, MA, USA) and ASET 2.0 (Sciencewares, Falmouth, MA, USA) and iFluxes 1.0 (YoungerUSA) software (Kochian *et al.*, 1992). Grafted plants were treated with 75 mM NaCl for 24 h, leading to significant accumulation of Na^+^ in roots and hypocotyls and activation of the Na^+^ efflux system. The roots and hypocotyls from control and salt-treated plants were then rinsed with distilled water and transferred to the measuring solution containing very little salt (0.1 mM KCl, 0.1 mM CaCl_2_, 0.1 mM MgSO_4_, 0.1 mM NaCl, 0.3 mM MES, pH 6.0). Plant specimens were immobilized in the middle of poly-L-lysine-coated coverslips (2 cm×2 cm) in the measuring chamber. Net fluxes were measured after 30 min (for roots) and 15 min (for hypocotyls) equilibration in low-Na^+^ solution. The measuring sites in hypocotyl were 1 cm above or below the grafting union. Before testing, the upper part of the seedling was removed by a razor blade to expose the xylem vessel (deep colored area indicated in Supplementary Fig. S3). The measuring site in root was 400 μm from the root tip (see Supplementary Fig. S3), which corresponds to the elongation zone and in which a vigorous efflux of Na^+^ has been observed in our previous study (Lei *et al.*, 2014). The magnitude of steady-state ion fluxes was calculated by data recorded over a 240 s period (Supplementary Fig. S4). The glass micropipettes and measuring solutions were prepared as previously described (Lei *et al.*, 2014).

### Determination of transpiration rate and stomatal conductance

The second recently expanded leaves were selected for the determination of transpiration rate and stomatal conductance with an open gas exchange system (Li-6400, Li-Cor, Inc., Lincoln, NE, USA). The assimilatory chamber was controlled to maintain the leaf temperature at 28 °C, CO_2_ concentration at 360 μmol mol^−1^, and photosynthetic photon-flux density at 600 μmol m^−2^ s^−1^. Five replicate plants per treatment were measured between 8:30 and 11:30 AM.

### Determination of relative water content

The relative water content (RWC) of leaves and roots was calculated as described by Weatherley (1950).

### Visualization of H_2_O_2_ in root using fluorescent dye

Confocal laser scanning microscopy (Leica TCS-SP2, Leica Microsystems GmbH, Wetzlar, Germany) was used to visualize H_2_O_2_ accumulation in plant roots *in vivo*. Roots from two grafted combinations were incubated in the reaction buffer containing 10 mM Hepes–NaOH (pH 7.5) and 10 μM 2′,7′-dichlorodihydrofluorescein diacetate (H_2_DCF-DA; Invitrogen) for 20 min at 30 °C. Thereafter, the roots were washed with the HEPES–NaOH buffer (pH 7.5) and fluorescence measurements conducted. The dye excitation was at 488 nm; emitted light was detected at 522 nm.

### Isolation of the plasma membrane vesicles and determination of NADPH oxidase and H^+^-ATPase activities

Root plasma membrane vesicles were isolated using a two-phase aqueous polymer partition system (Xia *et al.*, 2009). The NADPH-dependent O_2_--generating activity in isolated plasma membrane vesicles was determined by the protocol described previously (Zhou *et al.*, 2014). The H^+^-ATPase activity was determined by measuring the release of inorganic phosphate (P_i_) (Kłobus and Janicka-Russak, 2004) and expressed as the difference between the activities measured in the absence and presence of Na_3_VO_4_.

### Total RNA extraction and gene expression analysis

Total RNA was isolated from the seedling roots using TransZol reagent (TransGen Biotech, Inc., Beijing, China) in accordance with the manufacturer’s protocol. After extraction, the total RNA was dissolved in the diethylpyrocarbonate-treated water. The cDNA template for the quantitative real-time PCR (qRT-PCR) was synthesized from 1 μg of total RNA using HiScript II Q Select RT SuperMix for qPCR (Vazyme, Piscataway, NJ, USA).

For qRT-PCR analysis, we amplified the PCR products in triplicate by using 1×Top Green qPCR SuperMix (TransGen Biotech, Inc., Beijing, China) in 10 μl qRT-PCR assays. The PCR was performed using the ABI 7000 machine (Applied Biosystems), and the cycling conditions consisted of denaturation at 94 °C for 30 s, followed by 40 cycles of denaturation at 95 °C for 5 s, annealing at 55 °C for 15 s, and extension at 72 °C for 15 s. The specific primers (Table 1) were designed based on published mRNA of *Cucurbita moschata* on the Cucurbit Genomics Database (http://cucurbitgenomics.org) using Primer 5 software. The relative gene expression was determined as previously described by Livak and Schmittgen (2001).

**Table 1.** Gene-specific primers designed for qRT-PCR

Grafted combinations	Gene	Forward primer	Reverse primer	Genomics Database accession
C/C	*PMA*	GGCTGGTGTAGTTTGGA	CATAGTCTTTCTTGGTCGTA	Csa1G423270
	*SOS1*	CCAACGGAGTGGTAAA	AACAACGGAATCTGTAATC	Csa5G098980
	*RbohD*	AACAACATCAAGGACCAG	TCACCCAGTAGAAGTAAGC	Csa3G845500
	*RbohF*	AGCCAGAACATACAGGG	TTAGCCGTTAGGAGACAG	Csa4G050170
	*EF1a*	ACTGTGCTGTCCTCATTATTG	AGGGTGAAAGCAAGAAGAGC	Csa2G139820
C/P	*PMA*	TAGAGTGAAGCCATCTCC	CAAGCATAACGCCAGT	CmoCh11G003690
	*SOS1*	GGAGCCATTGGTTCGTC	GGTGCCTCGCAGTAAGT	CmoCh04G022490
	*RbohD*	ATGCCGAATACGAACC	ATTAGCACCACCATCACA	CmoCh14G010850
	*RbohF*	GTCATCTAACGAAACCTACA	TCCCATCCCTTAACCA	CmoCh04G007610
	*EF1a*	GCCTCAAACTCCAAGGATGA	GGCTCCTTCTCGAGTTCCTT	CmoCh08G009890

All primers were designed based on a published mRNA of *Cucurbita moschata* on the Cucurbit Genomics Database (http://cucurbitgenomics.org) using Primer 5 software. *EF1a* is the reference gene.

## Results

### Pumpkin-grafted cucumber was more tolerant than self-grafted cucumber

**Fig. 1.**
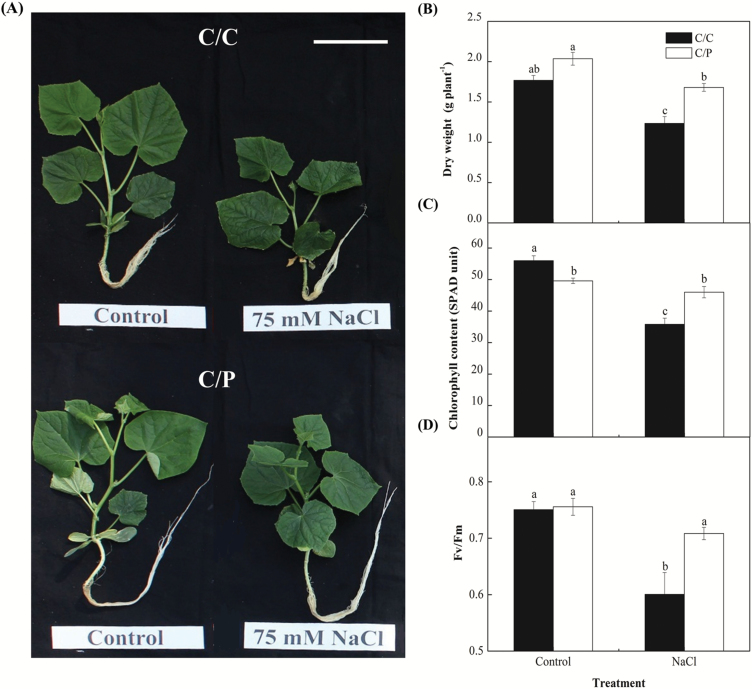
Effects of NaCl on the growth (A), dry weight (B), chlorophyll content (C) and photochemical efficiency (*F*_v_/*F*_m_) (D) of two grafted combinations, namely pumpkin-grafted cucumber (C/P) and self-grafted cucumber (C/C). Data are mean±SE (*n*=5). Columns with different letters are significantly different at *P*<0.05. Scale bar: 10 cm.

**Fig. 2.**
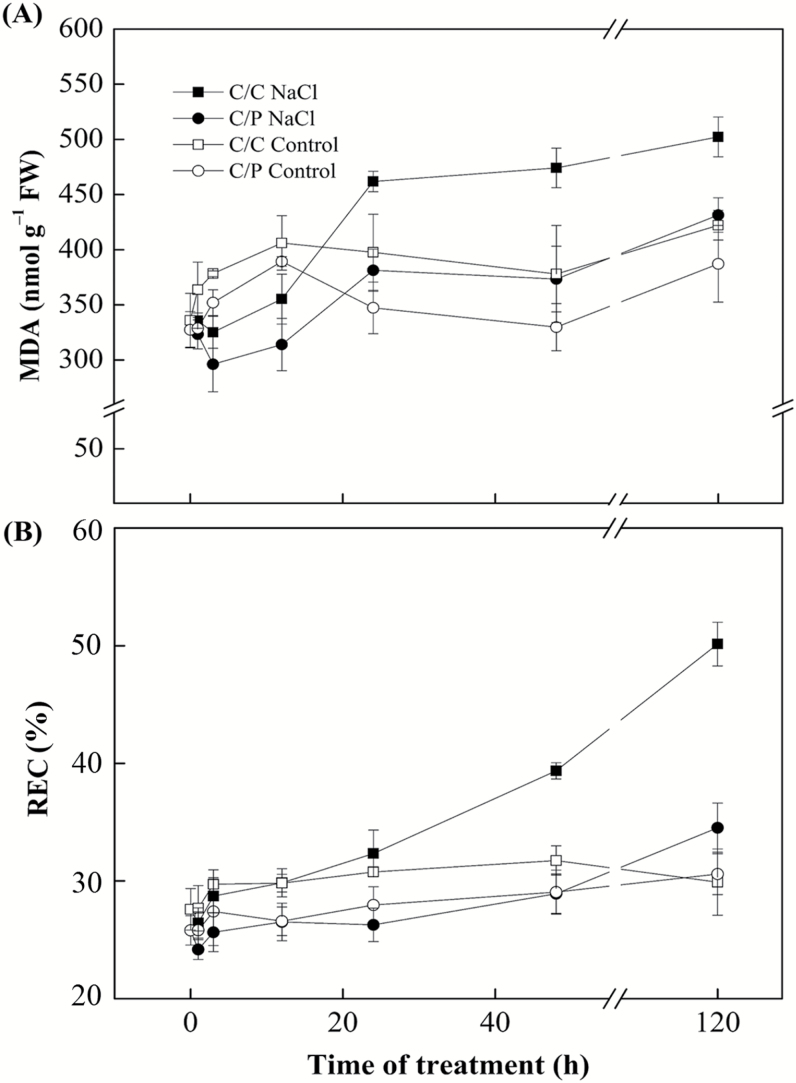
Effects of 75 mM NaCl treatments on malondialdehyde (MDA) content (A) and relative electric conductivity (REC; B) in the leaves of pumpkin-grafted cucumber (C/P) and self-grafted cucumber (C/C). Data are mean±SE (*n*=3).

Salt-induced biomass reduction was significantly stronger in the self-grafted (C/C) than in pumpkin-grafted (C/P) cucumber (Fig. 1A, B) after 5 d of salt treatment. Salt treatment had also caused a significant reduction in relative chlorophyll content (SPAD) and chlorophyll fluorescence in leaves of C/C (Fig. 1C, D). To confirm that the salt tolerance of C/P was higher than C/C, the level of MDA and relative electrical conductivity (REC) were measured in C/C and C/P. Salt stress increased MDA content and REC in C/C after 48 h, whereas in C/P plants, the increase in MDA content and REC was only observed after a prolonged treatment until 120 h (Fig. 2B), suggesting that C/P is indeed more tolerant of salt stress than C/C.

### Time-dependent kinetics of Na^+^ and H_2_O_2_ accumulation in salt-treated plants

**Fig. 3.**
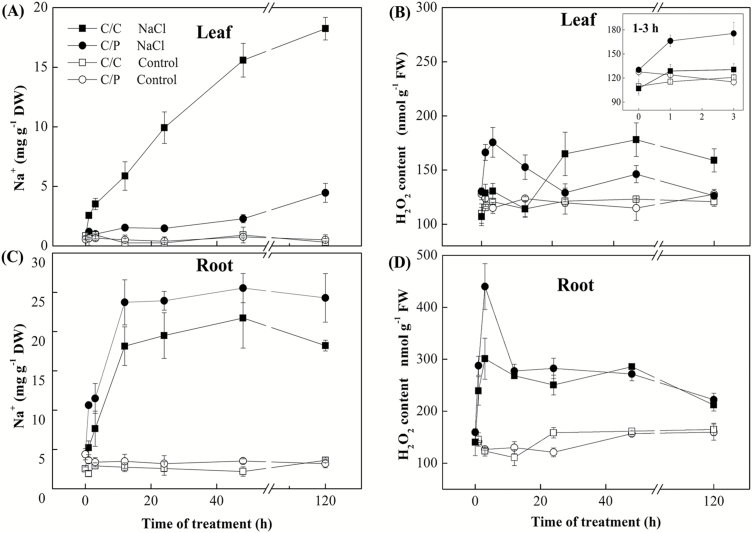
Effects of 75 mM NaCl treatments on accumulation patterns of Na^+^ and H_2_O_2_ in pumpkin-grafted cucumber (C/P) and self-grafted cucumber (C/C). Na^+^ and H_2_O_2_ contents were detected in leaves (A, B) and in roots (C, D). Data are mean±SE (*n*=3–5).

Na^+^ content in roots of both grafted combinations reached a plateau after about 12 h and then remained more or less constant (Fig. 3A, C), with C/P roots accumulating more Na^+^ compared with C/C roots (significant at *P*<0.05). In shoots, Na^+^ increased sharply in the self-grafted cucumber while in the pumpkin-grafted cucumber Na^+^ accumulation in the shoot became noticeable only after 48 h of salinity treatment. At the end of experiment, the Na^+^ concentration in leaves of C/C plants reached 18.2 mg g^−1^ DW, which was nearly 4 times higher than in the leaves of C/P.

**Fig. 4.**
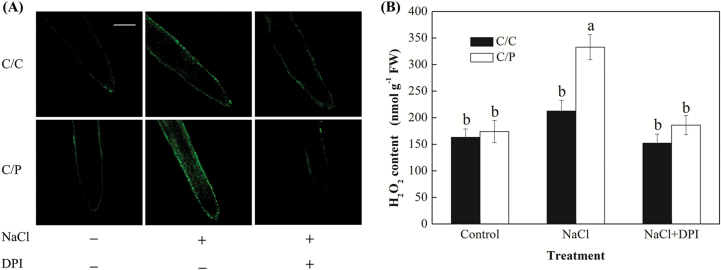
Effects of NaCl and DPI on the endogenous H_2_O_2_ level in the roots of pumpkin-grafted cucumber (C/P) and self-grafted cucumber (C/C). The plants were pretreated with DPI for 6 h and then transferred to Hoagland’s solution without DPI for 75 mM NaCl treatment. H_2_O_2_ levels were measured by using confocal fluorescence imaging from roots stained with H_2_DCF-DA. Scale bar: 100 μm. Data are mean±SE (*n*=3–5). Columns labeled with different letters are significantly different at *P*<0.05.

To determine the possible involvement of the H_2_O_2_ signal in stress tolerance, the levels of H_2_O_2_ in the self-grafted cucumber and the pumpkin-grafted cucumber were measured. The result indicated that the H_2_O_2_ content was rapidly elevated in roots of both grafted combinations and reached a peak at 3 h. Then H_2_O_2_ levels decreased during the period between 3 and 12 h but remained elevated for at least 48 h after commencement of the treatment (Fig. 3D). While the final H_2_O_2_ concentrations were not different between two grafting combinations, the NaCl-induced peak in H_2_O_2_ production was twice as high in C/P roots compared with their C/C counterparts (Fig. 3D). Similar results were reported when H_2_O_2_ content in root was visualized using the H_2_DCF-DA fluorescence probe (Fig. 4). Here, NaCl treatment caused a rapid increase in H_2_DCF-DA-dependent fluorescence in the roots of C/P, but not in C/C (Fig. 4). In leaves, stress-induced H_2_O_2_ increase was observed in C/P after 3 h, whereas in C/C plants, H_2_O_2_ increase was only observed after a prolonged treatment of 24 h (Fig. 3B).

### Effects of DPI on Na^+^ transport and accumulation in grafted plants

**Fig. 5.**
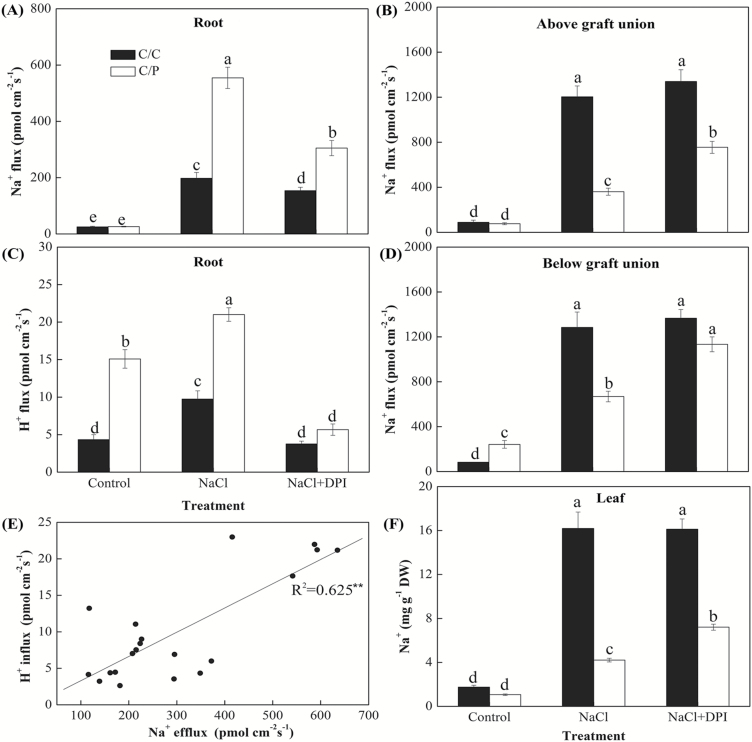
Net Na^+^ and H^+^ fluxes measured from root and shoot tissues of grafted plants using the non-invasive micro-test technology (NMT). (A, C) Net Na^+^ and H^+^ fluxes measured from the apical region of plant roots. (B, D) Net Na^+^ fluxes measured from the position 1 cm above (B) or below (D) graft union. Data are mean±SE (*n*=5 biological replicates). (F) Na^+^ content in leaf of two grafted combinations. Columns with different letters are significantly different at *P*<0.05. (E) The correlation between Na^+^ flux and H^+^ fluxes in roots. Each point represents an individual root measured under salinity conditions.

An NADPH oxidase inhibitor, DPI, was used to investigate the potential role of the plasmalemma-based H_2_O_2_ production in regulating Na^+^ transport and its accumulation in roots and shoots. At the whole-plant level, the DPI pretreatment increased Na^+^ concentration in leaves of C/P by 71% (Fig. 5F) after 120 h of salinity treatment, compared with non-inhibitor treatment. At the same time, inhibition of the NADPH oxidase resulted in no significant change in Na^+^ accumulation in leaves of C/C plants (Fig. 5F).

We next assayed the role of NADPH oxidase-produced H_2_O_2_ in regulation of ionic relations in root (Fig. 5A, C) and stem (Fig. 5B, D) at the cellular level, by measuring effect of DPI on net ion fluxes in these tissues using the NMT technique. In the apical regions of the roots, a massive efflux of Na^+^ from roots was recorded in two grafting combinations following the transfer of salt-treated roots to low-Na^+^ (0.1 mM) solution (Fig. 5A). The mean rates were 555 and 198 pmol cm^–2^ s^–1^ for C/C and C/P, respectively. Notably, the DPI-pretreated C/P displayed 45% lower flux than treatment without DPI while the reduction was only 22% in roots of C/C (Fig. 5A). Salt-treated roots also displayed a net H^+^ influx in both grafting combinations (Fig. 5C). Higher H^+^ influxes have been found in C/P compared with C/C, regardless of salinity treatment. DPI pretreatment decreased net H^+^ fluxes by 61% and 73% in roots of C/C and C/P, respectively. A significant positive correlation (*R*^2^=0.625; *P*<0.05) was found between Na^+^ efflux and H^+^ influx in salt-treated roots (Fig. 5E), suggesting the possibility of Na^+^/H^+^ antiport.

As mentioned above, the higher salt tolerance in pumpkin-grafted cucumber was correlated to the restricted transport of Na^+^ from root to shoot. To clarify the process of Na^+^ transport from root to shoot, we have used the NMT technique to measure ion flux at two positions along the hypocotyl: above (Fig. 5B) and bellow (Fig. 5D) the graft union. In the tissue below the graft union, net Na^+^ efflux of 1284 pmol cm^–2^ s^–1^ was detected in the C/C combination while in C/P it was half (only 669 pmol cm^–2^ s^–1^) indicating a reduced Na^+^ flux by rootstock of pumpkin. When fluxes were measured above the graft union, the values were 1204 pmol cm^–2^ s^–1^ for C/C but only 361 pmol cm^–2^ s^–1^ for C/P. Notably, root pretreatment with DPI caused much more Na^+^ to be translocated from the root to the shoot in the positions of above and below graft union in C/P. By contrast, the same treatment did not result in any detectable changes in Na^+^ translocation to the shoot in C/C.

### Effects of DPI on the generation of H_2_O_2_ in roots

**Fig. 6.**
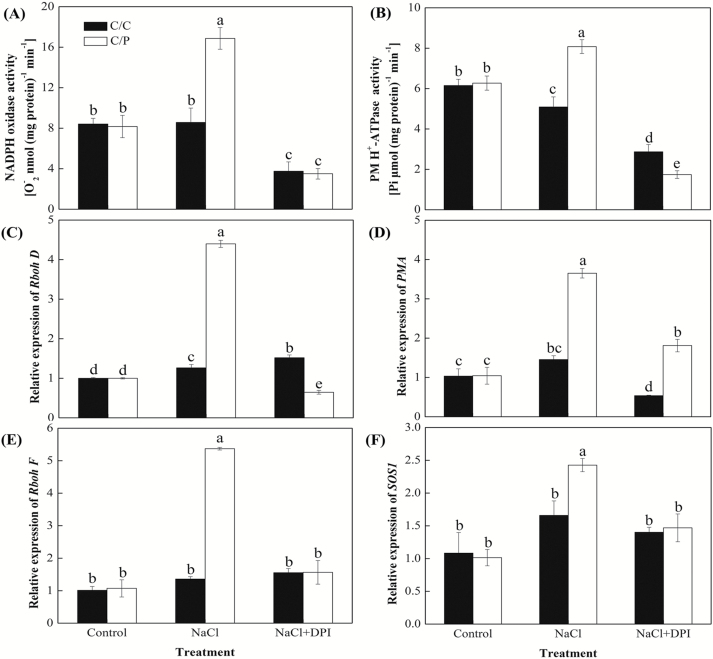
Effects of NaCl and DPI on the NADPH oxidase-based H_2_O_2_ generation (A, C, E) and Na^+^/H^+^ antiport system (B, D, F) in roots of pumpkin-grafted cucumber (C/P) and self-grafted cucumber (C/C) after 3 h of treatment. Data are mean±SE (*n*=3–5). Columns with different letters are significantly different at *P*<0.05.

To determine the relationship between the NADPH oxidase activity and salt-induced H_2_O_2_ signaling, we have measured endogenous H_2_O_2_ levels in salinized roots from the two grafted combinations. A analysis of the H_2_O_2_ content supported observation made by the confocal imaging (Fig. 4A). Pretreatment with DPI abolished the NaCl-induced H_2_O_2_ accumulations. Importantly, NaCl-induced NADPH oxidase activity was reduced in the DPI-pretreated plants at all time points measured (e.g. after both 3 h (Fig. 6A) and 24 h (see Supplementary Fig. S5) of salt treatment. The transcript level of *RbohD* and *RbohF* involved in the generation of NADPH oxidase were both rapidly elevated at 3 h in C/P after salt treatment, but only a small change was observed in C/C (Fig. 6C, E).

### DPI reduced Na^+^/H^+^ antiporter operation via depressing H^+^-ATPase activity

As DPI application had a concurrent effect on Na^+^ and H^+^ fluxes in roots, we hypothesized that a Na^+^/H^+^ antiport system has been a downstream target of the salt-induced H_2_O_2_ signal in roots. The change in the elevated PM H^+^-ATPase activities matched the change in the up-regulated expression of PMA in C/P, which might contribute to the H^+^-driven Na^+^ exclusion pathway (Fig. 5B, D, F). The inhibition of NADPH oxidase activity by DPI markedly decreased the PM H^+^-ATPase activities in C/C and C/P (Fig. 6B).

### Effects of DPI on shoot transpiration and H_2_O_2_ level in leaves

**Fig. 7.**
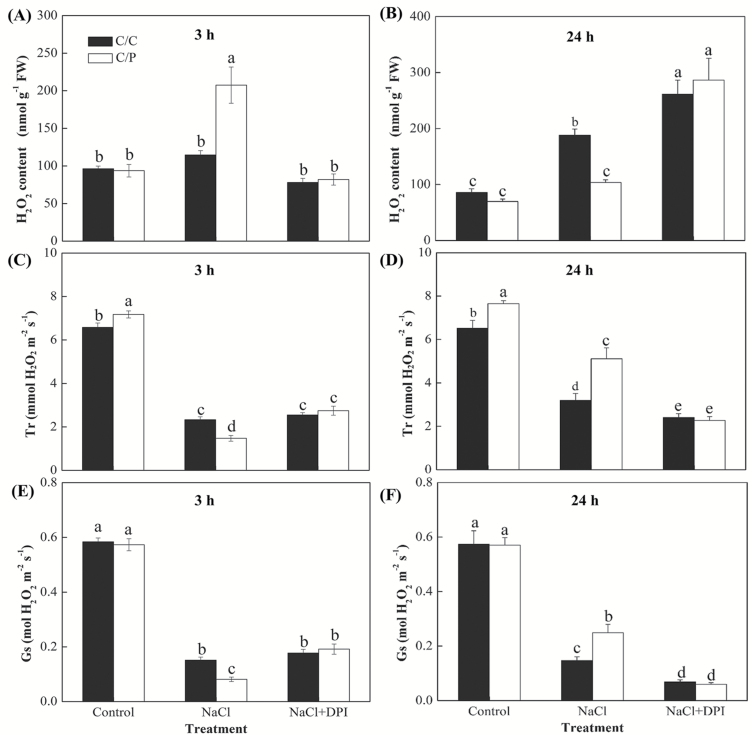
Effects of NaCl and DPI on the leaf H_2_O_2_ contents, leaf transpiration rate (*T*_r_) and stomatal conductance (*G*_s_) in pumpkin-grafted cucumber (C/P) and self-grafted cucumber (C/C) after 3 h (A, C, E) or 24 h (B, D, F) of treatment. Data are mean±SE (*n*=3–5). Columns with different letters are significantly different at *P*<0.05.

The root-to-shoot delivery of Na^+^ is affected not only by the rate of xylem Na^+^ loading but also by the transpiration lift that is conferred by the opening of stomata. In this study, NaCl treatment resulted in a rapid stomatal closure in both grafted combinations in the first 3 h. This closure was more pronounced in C/P than in C/C (80% *versus* 65% reduction, respectively). The observed trend for the transpiration rate was similar to the trend for the stomatal conductance (Fig. 7C, E). After 24 h of salt treatment, stomatal conductance (*G*_s_) and leaf transpiration rate (*T*_r_) recovered in both grafting combinations but were still lower than those in control (Fig. 7D, F), and the C/P maintained higher *G*_s_ and *T*_r_ values than C/C. After 3 h of salt treatment, significantly elevated H_2_O_2_ levels were only found in the leaves of C/P, and this increase in the leaf H_2_O_2_ could be inhibited by DPI pretreatment in the roots (Fig. 7A). In contrast, a significant increase in the H_2_O_2_ level was observed in the leaves of C/C only after 24 h of salt stress (Fig. 7B). Interestingly, compared with non-DPI treatment, pretreatment with DPI caused higher H_2_O_2_ levels in both grafted combinations after 24 h of salt treatment (Fig. 7B).

## Discussion

Grafting is an effective way to increase salt tolerance of plants. While previous studies investigated the underlying mechanisms behind the beneficial effects of grafting from the point of view of altered ion homeostasis (Edelstein *et al.*, 2011), root-derived hormones (Albacete *et al.*, 2009) and the antioxidant system (He *et al.*, 2009), the signal transduction aspects of grafting (and, specifically, the role of root-derived H_2_O_2_ signals) have never been put in the spotlight. Hydrogen peroxide signaling is known to be important for the acclimation to salt stress conditions (Wang *et al.*, 2013; Hossain and Dietz, 2016). In addition, hydrogen peroxide is one of the known signaling molecules that has an ability to travel long distances (Baxter *et al.*, 2014; Gilroy *et al.*, 2014) and may potentially enable communication between remote plant tissues and organs (Mittler and Blumwald, 2015). Therefore, the grafted plant is a good model for understanding ROS function between root and shoot. Here, two grafted combinations were used to clarify the role of the root-sourced H_2_O_2_ in plant response to salt stress. The results indicate that NADPH-generated root H_2_O_2_ signals control at least two processes that are essential for plants to handle the salt load. One of them is regulation of Na^+^ exclusion from roots and the other is rapid stomatal closure upon stress onset (Figs 5 and 7).

### H_2_O_2_ signal involved in the Na^+^ exclusion process in pumpkin-grafted plants

As only a small proportion of Na^+^ can be retrieved from the shoot and moved back to the root via the phloem in plants (Munns and Tester, 2008; Lei *et al.*, 2014), the key factor that determine the Na^+^ accumulated in scion is the restrictive ability of rootstock to load Na^+^, as evident from the comparison of different grafting combinations (Zhu *et al.*, 2008; Huang *et al.*, 2010; Edelstein *et al.*, 2011). This notion was further supported in this study. Exposure to salt stress results in increased expression of both *SOS1* transcripts (Shi *et al.*, 2000) and *SOS1*-mediated Na^+^/H^+^ exchanger activity in root epidermis (Sun *et al.*, 2009). Here we showed that C/P grafted plants were more efficient in effluxing Na^+^ from roots compared with C/C plants (Fig. 5A). A strong correlation between H^+^ influx and Na^+^ efflux (Fig. 5E) and a reported sensitivity of the measured Na^+^ efflux to amiloride (Cuin *et al.*, 2011) suggest that the Na^+^ exclusion in the salt-stressed plants roots is likely the result of an active Na^+^/H^+^ antiport across the PM. This result is consistent with the earlier findings from non-grafted cucumber and pumpkin (Lei *et al.*, 2014) that indicated higher Na^+^ exclusion capacity in pumpkin roots.

Many reports have demonstrated that H_2_O_2_ is a key signaling molecule involved in regulation of Na^+^ transport under salt stress. Among other sources, H_2_O_2_ is generated by the plasma membrane-located NADPH oxidase that is encoded by *RbohD* and *RbohF* (Xie *et al.*, 2011; Hossain and Dietz, 2016). DPI blocked salinity-induced H_2_O_2_ production and reduced salinity tolerance in Arabidopsis (Leshem and Seri, 2007), *Oryza sativa* (Wang *et al.*, 2013) and *Populus euphratica* (Sun *et al.*, 2010). Here we found a pronouncedly decrease in Na^+^ efflux that has mirrored a reduced H_2_O_2_ content and NADPH oxidase activity in C/P roots when pretreated with DPI (Fig. 6A, C, E).

The relationship between the SOS pathway and NADPH oxidase-mediated H_2_O_2_ signaling has remained elusive until now. It has been suggested that the NADPH oxidase may operate as a salt sensor in plants in tandem with Ca^2+^-permeable channels (Shabala *et al.*, 2015). According to this model, the plant plasma membranes harbor various non-selective cation channels (NSCCs), which are permeable to Ca^2+^ and may be activated by both ROS and membrane depolarization (Demidchik and Maathuis, 2007). As a second messenger, Ca^2+^ could bind to SOS3, which functions in the sensing the Ca^2+^ signal and contributes to PM Na^+^/H^+^ antiporter (SOS1) activation and regulation of cellular Na^+^ homeostasis. NADPH oxidase-mediated H_2_O_2_ accumulation is also critical to *SOS1* mRNA stability (Chung *et al.*, 2008). Sun *et al.* (2010) found that reduction in H_2_O_2_ production caused by DPI led to decreased Na^+^ efflux and H^+^ influx, and the Ca^2+^ concentration in the cytoplasm also decreased. Our work reported here suggests that another factor contributing to the stronger Na^+^ efflux in C/P roots is the higher activity of PM H^+^-ATPase, which sustains an H^+^ gradient to drive Na^+^/H^+^ antiport across the PM (Fig. 6B, D and Supplementary Fig. S6). This is consistent with the previous observations in non-grafted pumpkin roots, where vanadate (a PM H^+^-ATPase inhibitor) treatment concurrently decreased both Na^+^ efflux and H^+^ influx (Lei *et al.*, 2014). The possible reason for this may be found in the fact that PM H^+^-ATPase activation may be also mediated by Ca^2+^. It has been reported that the activity of PM H^+^-ATPase was dependent on the Ca^2+^ concentration in the cytosol (Zhang *et al.*, 2007) and the interaction of [Ca^2+^]_cyt_ and a calcium-dependent protein kinase regulated the PM H^+^-ATPase in response to fungal elicitors (Lino *et al.*, 1998). As DPI pretreatment decreased both the NADPH oxidase and H^+^-ATPase activity in roots of both grafting combinations, it appears that the root-derived H_2_O_2_ signaling pathway is shared by both plants (i.e. cucumber and pumpkin), but with a different efficiency.

It is obvious that the Na^+^ exclusion mechanism is just one of many strategies employed by plants to deal with the salt load. Other mechanisms such as storage of excess Na^+^ in vacuoles and restrictions on Na^+^ loading into the root stele have also been reported in pumpkin roots (Lei *et al.*, 2014). These might be the reason why C/P plants possessed a higher Na^+^ efflux but still had more Na^+^ accumulated in their roots compared with C/C (Fig. 3C). Our previous study also found that some pumpkin genotypes stored a vast amount of Na^+^ in the stems (Niu *et al.*, 2017) and the genes involved in the Na^+^ compartmentation process (*HKT*, *NHX*) exhibited even higher expression levels in the hypocotyl than in the root (data not published). Using the xylem saps collected from below or above the grafting union, a significant decrease in Na^+^ concentration has been found in pumpkin-grafted cucumber but nearly no difference in the self-grafted cucumber, suggesting that the grafting union is a barrier for Na^+^ transport when pumpkin is used as a rootstock (Huang *et al.*, 2013). In the present study, the NMT data are consistent with our previous result for the xylem sap (Fig. 5B, D).

### Root-sourced H_2_O_2_ signals trigger rapid stomatal closure in the shoot

In recent years, H_2_O_2_ has firmly established itself as an important second messenger mediating the broad range of adaptive plant responses (Mittler *et al.*, 2011; Baxter *et al.*, 2014). H_2_O_2_ production is highly tissue-specific and possesses a rather complex kinetics. In *Cakile maritima*, the H_2_O_2_ concentration reached a peak within 4 h of salt stress and rapidly declined afterwards, while H_2_O_2_ continued to rise in Arabidopsis during the first 72 h after salt treatment (Ellouzi *et al.*, 2011). Xie *et al.* (2011) also found that mild salt stress causes a rapid and transient accumulation of ROS in Arabidopsis peaking after 1 h followed by a second oxidative burst after about 6 h. These and similar findings have led to the suggestion that H_2_O_2_ ‘signatures’ may operate in plant signaling networks (Bose *et al.*, 2014), in addition to well-known cytosolic calcium ‘signatures’ (Dodd *et al.*, 2010). The current work supports this hypothesis and demonstrates that early root-derived H_2_O_2_ signals are essential for early stomata closure in grafted C/P combination but is lacking in C/C plants.

In a longer period, accumulation of large amounts of Na^+^ in leaves of C/C (Fig. 3A) forced the plant to close the stomata to reduce the amount of Na^+^ delivered from root to shoot via the transpiration flow. In contrast, the pumpkin-grafted plants, C/P, gain advantage in restricting a large proportion of Na^+^ to roots and hypocotyls and accumulating less Na^+^ in the leaves (Fig. 3A), making such stomatal closure less essential later on (Fig. 7D, F). DPI-treated C/P impaired the Na^+^/H^+^ antiport system located in C/P roots (Fig. 6), which led to an overaccumulation of Na^+^ in shoots (Fig. 4) and required a more restricted transpiration rate in C/P (Fig. 7D), to protect the photosynthetic system from the ionic stress. However, in the first several hours of salinity, the Na^+^ in leaves did not reach a toxic level in either of the two combinations (less than 5 mg g^−1^; Fig. 3A). Thus, a rapid closure of stomata in the early period in C/P plants is unlikely to be driven by the need to restrict Na^+^ delivery to the shoot but instead may be related to an early signal induced by roots to deal with osmotic stress, reducing water loss and maintaining the plant’s hydration status (see Supplementary Figs S7B and S8).

The obvious question arising from this data is, why are C/P plants able to sense and signal salt stress faster than C/C plants? Given that such signaling was causally related to the root RBOH-dependent H_2_O_2_ production, this points to NADPH acting as a tentative sodium sensor, and its more efficient operation (higher sensitivity) in pumpkin roots.

Our current knowledge of how salt stress is sensed by plant tissues is severely limited (Maathuis, 2014; Shabala *et al.*, 2015), and it is highly likely that more than one of the sensory mechanisms may operate in the same cell at the same time, encoding specific information on stress severity, and sharing some common downstream signaling pathway(s). NADPH oxidase has been suggested to be one of these (Shabala *et al.*, 2015). NADPH oxidases are activated by salt stress, at both the transcriptional and the functional level (Xie *et al.*, 2011), and plants lacking functional *AtrbohD* and *AtrbohF* genes showed increased hypersensitivity to salinity (Ma *et al.*, 2012), suggested that the NADPH oxidase may also operate as a salt sensor in plants. The model also assumes that NSCCs are located in the immediate proximity of the NADPH oxidase, forming a microdomain in a lipid raft. The onset of the salt stress will lead to a rapid (within seconds) membrane depolarization by 50–80 mV (Shabala *et al.*, 2005; Jayakannan *et al.*, 2015; Chakraborty *et al.*, 2016), resulting in the instantaneous activation of NSCCs and causing a rapid elevation in the cytosolic Ca^2+^. This elevation will result in a rapid activation of NADPH oxidase and a concurrent increase in ROS accumulation in the apoplastic space. These ROS will further activate NSCCs and amplify stress-induced Ca^2+^ and ROS transients via self-amplification loops. This self-amplification loop seems to be more efficient in C/P than in C/C grafted plants. Future studies should reveal the molecular mechanisms behind this regulation, as well as interaction of the root RBOH-derived H_2_O_2_ signals with other signals propagating between roots and shoots in salt-stressed plants (Baxter *et al.*, 2014; Gilroy *et al.*, 2014; Shabala *et al.*, 2016).

## Conclusion

In summary, we have discovered that the root RBOH-dependent H_2_O_2_ production operates as an early signal to regulate salt tolerance of grafted cucumber through Na^+^ exclusion and stomatal closure. This mechanism can alleviate the salt-induced damage in the rootstock-grafted cucumber plants.

## Supplementary data

Supplementary data are available at *JXB* online.

Fig. S1. Effects of various NaCl concentrations on the growth of the pumpkin-grafted cucumber (C/P) and a self-grafted cucumber (C/C).

Fig. S2. Effects of various NaCl concentrations on H_2_O_2_ accumulation in roots of two grafted combinations, namely the pumpkin-grafted cucumber (C/P) and a self-grafted cucumber (C/C).

Fig. S3. The sampling positions for ion fluxes test by the non-invasive micro-test technology (NMT).

Fig. S4. A four-minute continuous flux recording was conducted using NMT from roots of plants from two grafted combinations, namely the pumpkin-grafted cucumber (C/P) and a self-grafted cucumber (C/C).

Fig. S5. Effects of NaCl and DPI on the NADPH oxidase-based H_2_O_2_ generation and Na^+^/H^+^ antiport system in roots of the pumpkin-grafted cucumber (C/P) and a self-grafted cucumber (C/C) after 24 h of treatment.

Fig. S6. Effect of 75 mM NaCl treatment on the time dependence of *SOS1* (A) and *PMA* (B) transcriptions in roots of the pumpkin-grafted cucumber (C/P) and a self-grafted cucumber (C/C).

Fig. S7. Effect of grafting on kinetics of plant wilting and relative water content for the pumpkin-grafted cucumber (C/P) and a self-grafted cucumber (C/C) treated with 75 mM NaCl.

Fig. S8. Effect of NaCl and DPI on the stomatal aperture in the detached abaxial epidermal strips from the pumpkin-grafted cucumber (C/P) and a self-grafted cucumber (C/C) after 3 h of treatment.

## Supplementary Material

Supplementary Figures S1-S8
