# Supplementary material for: Root respiratory burst oxidase homologue-dependent H2O2 production confers salt tolerance on a grafted cucumber by controlling Na+ exclusion and stomatal closure
Source: J Exp Bot. 2017 Nov 14;69(14):3465–76. doi: 10.1093/jxb/erx386 (PMC6009698; doi:10.1093/jxb/erx386)
Supplement: Supplementary Figures S1-S8 [file erx386_suppl_supplementary-figures-s1-s8.pdf]

Supplemental Figures

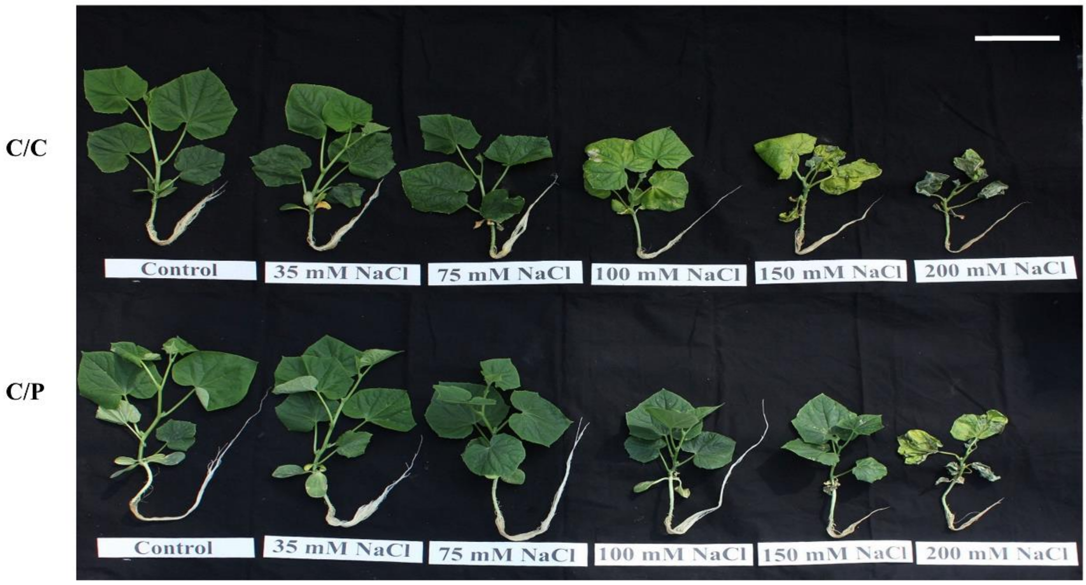

Supplementary Fig. S1. Effects of various NaCl concentrations on the growth of the pumpkin-grafted cucumber (C/P) and a self-grafted cucumber (C/C). NaCl was added to the medium for 5 days. Bar =10 cm.

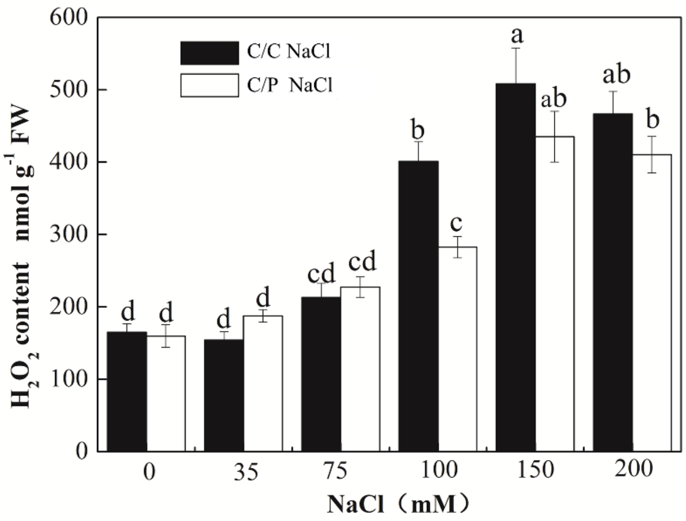

Supplementary Fig. S2. Effects of various NaCl concentrations on H_2O_2 accumulation in roots of two grafted combinations, namely the pumpkin-grafted cucumber (C/P) and a selfgrafted cucumber (C/C). NaCl was added to the medium for 5 d. Data are mean ± SE (n = 3 to 5). Columns with different letters are significantly different at $P< 0.05$. Bar =10 cm.

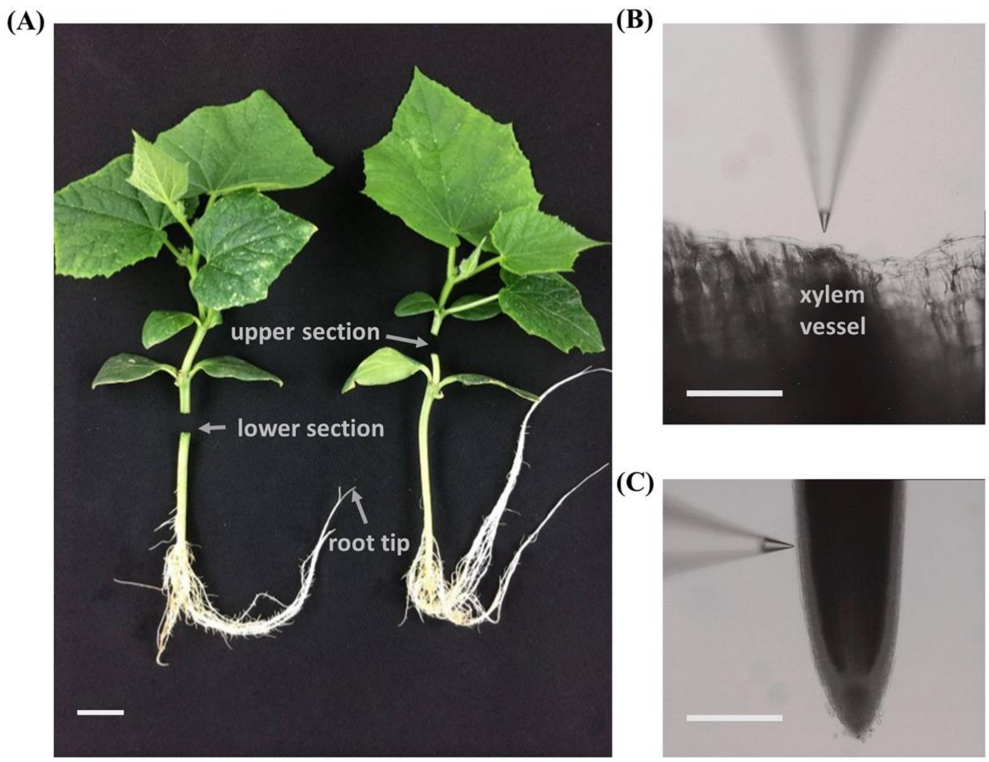

Supplementary Fig. S3. The sampling positions for ion fluxes test by the non-invasive micro-test technology (NMT). (A) The measuring sites in the hypocotyl were 1 cm above (upper section) or below (lower section) the grafting union. Bar = 5 cm. (B, C) microelectrode positioning for ion flux measurements. (B) For measurements from the hypocotyls, the upper of the seedling was removed by a razor to expose the xylem vessel (deep color area). Bar = 200 μm. (C) For root measurements, electrodes were positioned 400 μm from the tip. Bar = 200 μm.

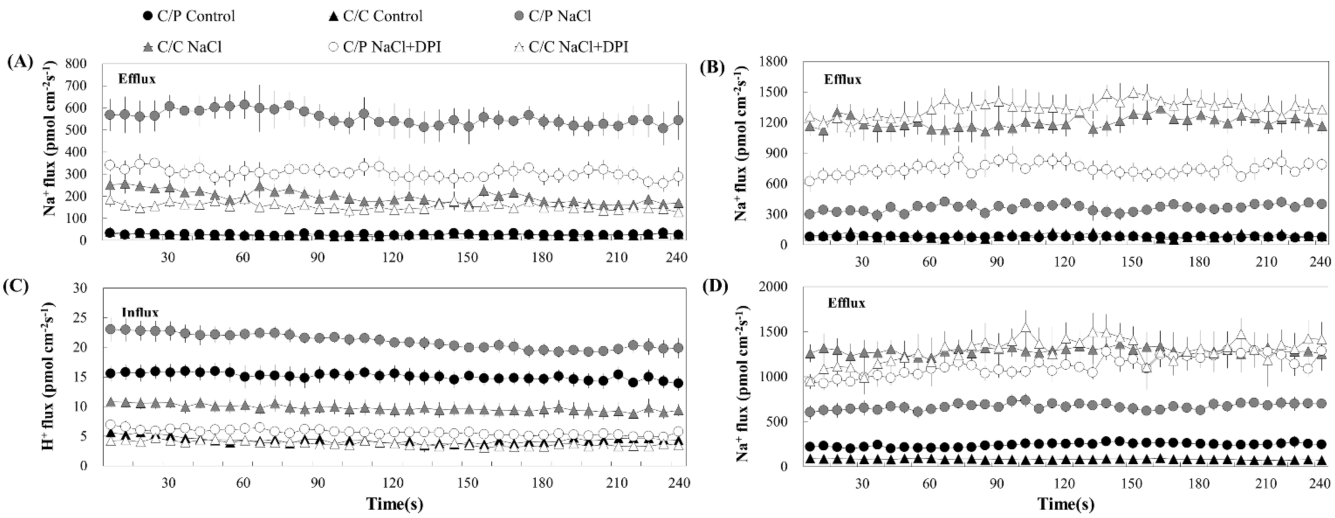

Supplementary Fig. S4. A four-minute continuous flux recording was conducted using NMT from roots of plants from two grafted combinations, namely the pumpkin-grafted cucumber (C/P) and a self-grafted cucumber (C/C). Each point represents the mean of five individual roots and bars represent the SE of the mean. The mean ion fluxes in the root tips (A), and sections located 1 cm above (B) or below (D) the grafting union, are shown. Columns labeled with different letters are significantly different at $P < 0.05$.

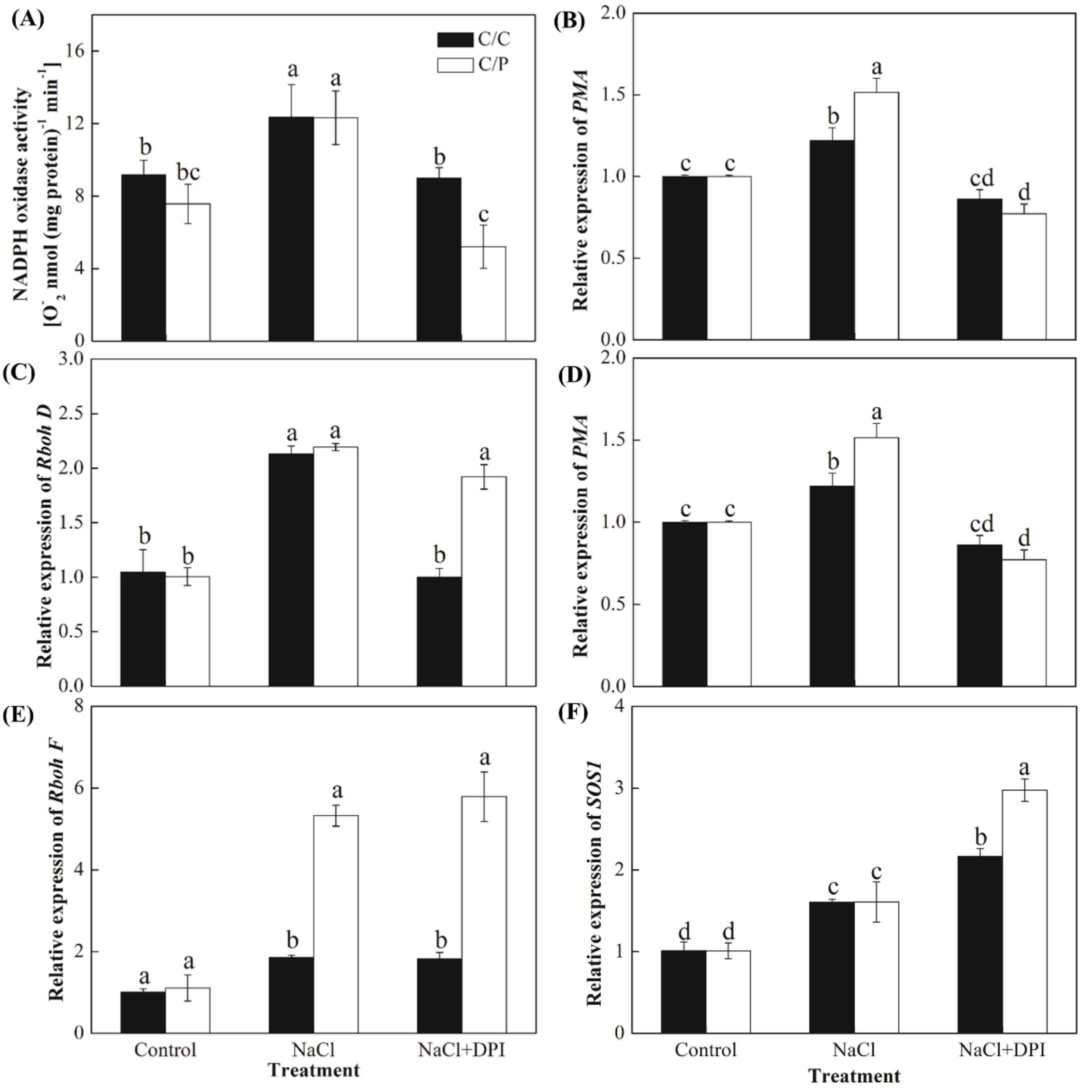

Supplementary Fig. S5. Effects of NaCl and DPI on the NADPH oxidase-based H_2O_2 generation (A, C and E) and Na^+/H^+ antiport system (B, D and F) in roots of the pumpkingrafted cucumber (C/P) and a self-grafted cucumber (C/C) after 24 h of treatment. Data are mean ± SE (n = 3 to 5). Columns with different letters are significantly different at $P< 0.05$.

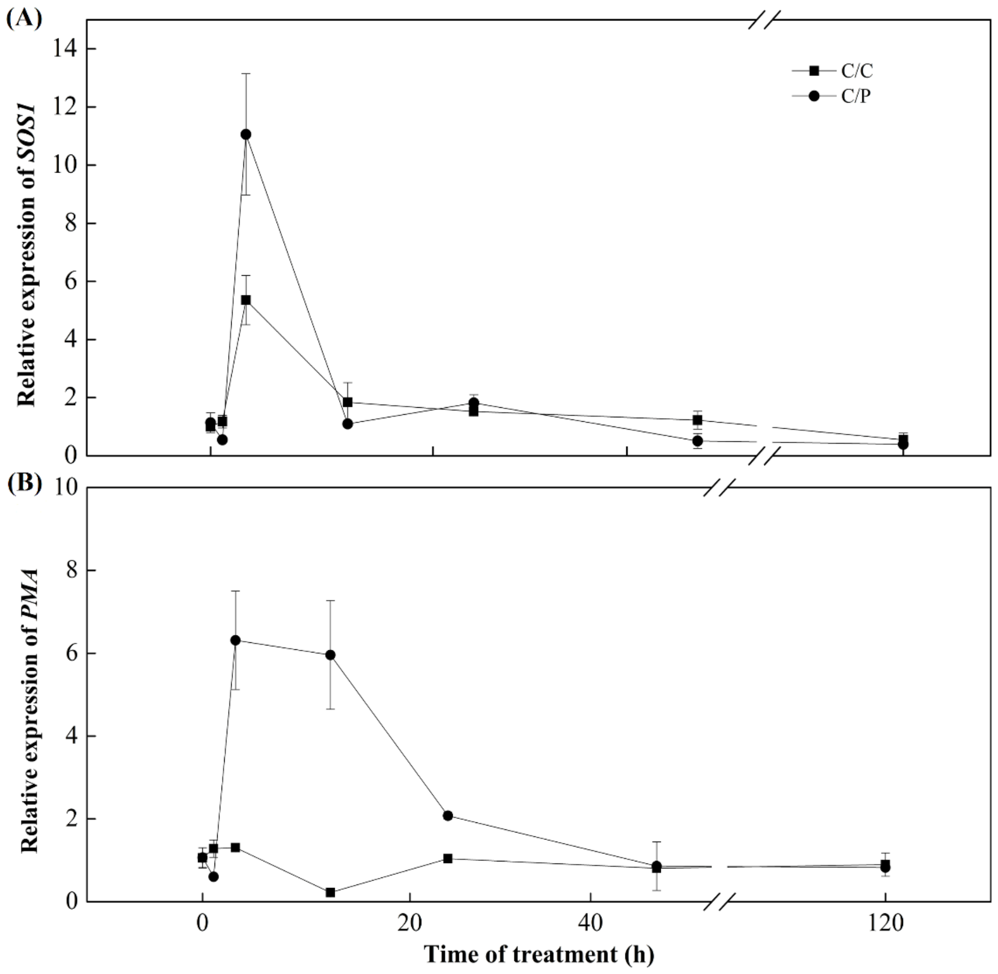

Supplementary Fig. S6. Effect of 75 mM NaCl treatment on the time dependence of *SOS1*(A) and *PMA* (B) transcriptions in roots of the pumpkin-grafted cucumber (C/P) and a selfgrafted cucumber (C/C). Data are mean ± SE (n = 3).

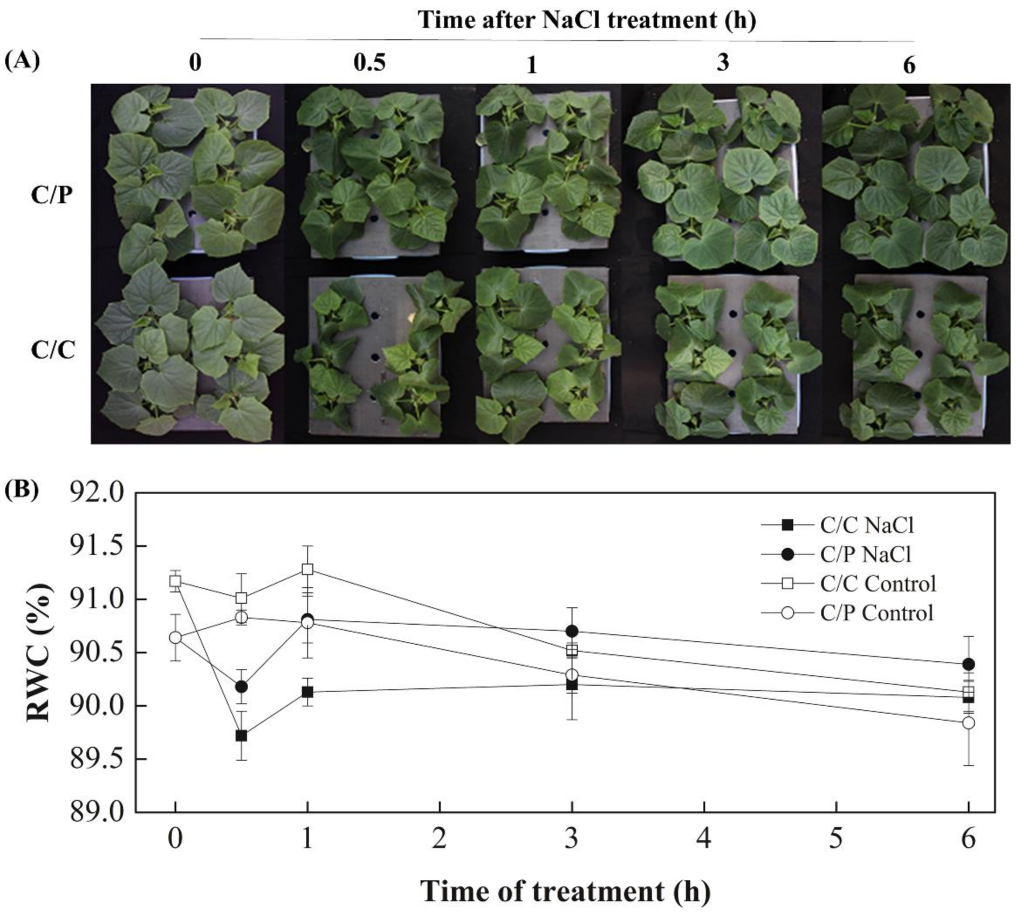

Fig. S7. Effect of grafting on kinetics of plant wilting and relative water content for the pumpkin-grafted cucumber (C/P) and a self-grafted cucumber (C/C) treated with 75 mM NaCl.

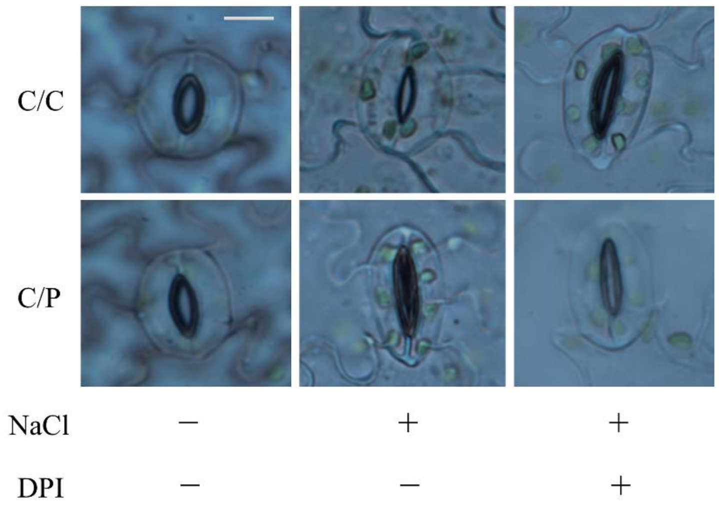

Supplementary Fig. S8. Effect of NaCl and DPI on the stomatal aperture in the detached abaxial epidermal strips from the pumpkin-grafted cucumber (C/P) and a self-grafted cucumber (C/C) after 3 h of treatment. Scale bar = 25 µm. Stomatal aperture was measured as following: epidermal strips from second recently expanded leaves were peeled from the abaxial surface with forceps, floated on buffer [30 mM KCl, 10 mM 2-(N-morpholino)-ethanesulfonic acid (MES), pH 6.15]. Then the stomatal aperture was immediately measuredusing a microscope (BX41, Olympus Co., Tokyo, Japan).
